# The outcomes of microvascular decompression for primary trigeminal neuralgia: insights from a single-center experience and technical advancements

**DOI:** 10.3389/fsurg.2024.1378717

**Published:** 2024-05-22

**Authors:** Chingiz Nurimanov, Iroda Mammadinova, Karashash Menlibayeva, Assylbek Kaliyev, Yerbol Makhambetov, Serik Akshulakov

**Affiliations:** ^1^ Vascular and Functional Neurosurgery Department, National Centre for Neurosurgery, Astana, Kazakhstan; ^2^ Hospital Management Department, National Centre for Neurosurgery, Astana, Kazakhstan

**Keywords:** trigeminal neuralgia, neurovascular conflict, microvascular decompression, long-term outcomes, technical note

## Abstract

**Background:**

Microvascular decompression (MVD) remains the primary surgical treatment for trigeminal neuralgia due to its positive postoperative results. This study aims to evaluate the outcomes of patients with primary trigeminal neuralgia who underwent MVD. Additionally, the paper offers a detailed explanation of the surgical methodology of MVD employed at the neurosurgical hospital in Kazakhstan.

**Methods:**

The study involved 165 medical records of patients with trigeminal neuralgia who underwent MVD between 2018 and 2020. Out of these 165 patients, 90 (54.55%) were included in the final analysis and were further evaluated using the Barrow Neurological Institute pain intensity score. Various variables were analyzed, including age, sex, affected side, dermatomes, offending vessel, and surgical intervention type. Moreover, the surgical technique employed at the hospital was described.

**Results:**

The average follow-up period after the MVD procedure was 32.78 ± 9.91 months. The results indicated that out of the 90 patients, 80 (88.89%) achieved a good outcome as evidenced by BNI scores I and II. It was observed that patients with affected maxillary dermatomas and those with affected ophthalmic + maxillary dermatomas were more likely to experience fair + poor postsurgery BNI scores. On the other hand, patients with neurovascular conflicts involving the maxillary + mandibular dermatomas demonstrated good BNI scores (*p* = 0.01).

**Conclusions:**

The outcomes of MVD in patients with primary trigeminal neuralgia showed good BNI scores within this study population. The outcome depended on the affected dermatome of the trigeminal nerve with the vessel. Additionally, patient positioning, intraoperative management including small skin incisions, minimal craniotomy, and precise closure of the dura, as well as intraoperative neurolysis, may contribute to achieving good clinical and satisfactory post-surgery aesthetic outcomes.

## 1 Introduction

Trigeminal neuralgia (TN), also known as “tic douloureux”, is a severe syndrome characterized by sudden, paroxysmal episodes of pain originating in one or more branches of the trigeminal nerve. The disease was initially documented by Nicolas André in 1756. Characteristics of facial pain and treatment recommendations such as resting in a dark room, bathing in warm water, and drinking wine have also been observed in ancient texts written by Galen, Avicenna, and Aretaeus (1, 2).

The symptom of TN is excruciating facial pain, often elicited by specific stimuli such as tactile sensations, pressure on sensitive facial areas, speech, mastication, tooth brushing, or ingestion of hot or cold substances. These triggers precipitate sudden and severe pain episodes, leading to discomfort and functional impairment in daily activities (3, 4).

The annual incidence of TN is estimated at 27 cases per 100,000 person-years, with a higher frequency among females at 7.3 cases vs. 3.7 cases per 100,000 person-years in males. The peak occurrence of TN is between the ages of 50 and 60 (5, 6). TN may involve one or multiple divisions of the trigeminal nerve. While TN impacts a single branch, the second division is the most frequently affected among the branches.

Treatment of TN includes pharmacotherapy, percutaneous destructive procedures, gamma-knife radiosurgery, and MVD (7–9). Each treatment modality is carefully applied, considering patients' characteristics, and prioritizing procedural safety. The pharmacological treatment presents a challenge due to the side effects, impacting approximately 30%–40% of cases and exacerbating with prolonged usage. With disease progression, patients may necessitate increased medication doses, potentially resulting in the accumulation of toxic or sub-toxic levels (10, 11). Destructive interventions provide rapid pain alleviation, albeit accompanied by constrained long-term effectiveness and the imperative for repeated interventions. Furthermore, these procedures entail significant risks, including hypoesthesia, neuropathic and deafferentation pain, keratopathy, and potential risks associated with radiation exposure in gamma knife interventions (12, 13).

Among the available treatment options, MVD is the most frequently used method and demonstrates positive long-term outcomes with minimal complications and low rates of morbidity and mortality (7–9). The procedure was first performed by Walter Dandy in 1925 and was later modified by Jannetta in 1967 (14). It involves identifying and delicately dissecting the affected trigeminal nerve from the compressing blood vessels by placing artificial material, usually Gore-Tex, Teflon, or less commonly used auto-transplants, such as small pieces of muscle (14, 15). The main goal of MVD is to alleviate the pressure on the trigeminal nerve, reducing or eliminating the pain signals that cause TN.

Since its introduction, the MVD technique has undergone multiple refinements aimed at repositioning the vessels compressing the trigeminal nerve root. These variations have shown a significant success rate, providing long-term pain relief in about 70% of cases, on average (8, 16, 17). Considering the recommendations and modifications, this paper aims to assess the outcomes of patients with primary TN who underwent MVD, while also describing institutional experience with MVD for TN.

## 2 Materials and methods

**Figure 1:**
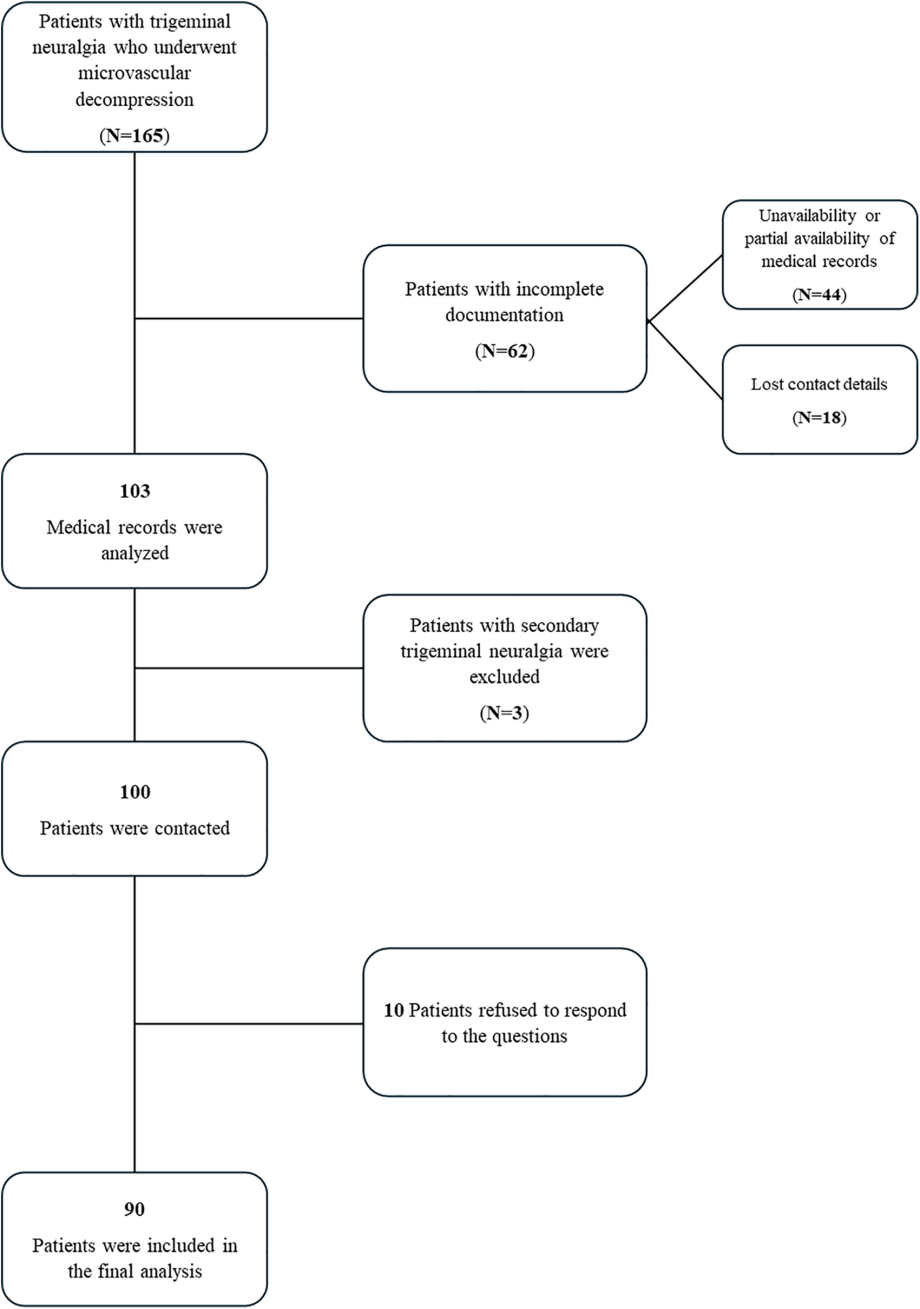
Patient exclusion and inclusion flow chart.

The retrospective study initially included 165 patients with TN who underwent MVD at the Vascular and Functional Neurosurgery Department, National Center for Neurosurgery, Astana, Kazakhstan, between January 2018 and December 2020. Patients with a confirmed diagnosis of primary symptomatic TN were included in the study. Patients with incomplete documentation (unavailability or partial availability of some medical records and lost contact details) due to the novel electronic system installed in the hospital (*N* = 62), patients' unwillingness to participate to respond (*N* = 10), and those diagnosed with secondary TN (*N* = 3) were excluded from the analysis resulting in a final sample size of 90 patients (Figure 1).

Medical records and radiological findings, as well as surgical protocols, were carefully reviewed. Radiological assessments using T2-weighted and FIESTA sequences on MRI scans were employed to detect neurovascular conflicts, providing a detailed examination of neurovascular dynamics (8).

### 2.1 Follow-up

The early postoperative evaluation was conducted by a neurologist within the hospital on the day following surgery. The neurologist assessed the postoperative condition of cranial nerve function, encompassing eye movement, facial motor and sensory function, hearing, swallowing, and phonation. Further follow-up was conducted through telephone interviews to assess the patient's condition. Pain intensity was measured using the Barrow Neurological Institute (BNI) pain intensity score.

### 2.2 Statistical analysis

Data was cleaned and coded using Microsoft Excel [Microsoft Office (Microsoft Corp., Redmond, Washington, USA)], and statistical analysis was performed on Stata 18 Standard Edition (StataCorp, College Station, Texas, USA). Descriptive statistics included reporting proportions, frequencies, and mean values ± standard deviation. Differences between variables were tested using Filcher's exact test. A *p*-value less than 0.05 was considered statistically significant.

## 3 Surgical technique

### 3.1 Patient positioning

**Figure 2:**
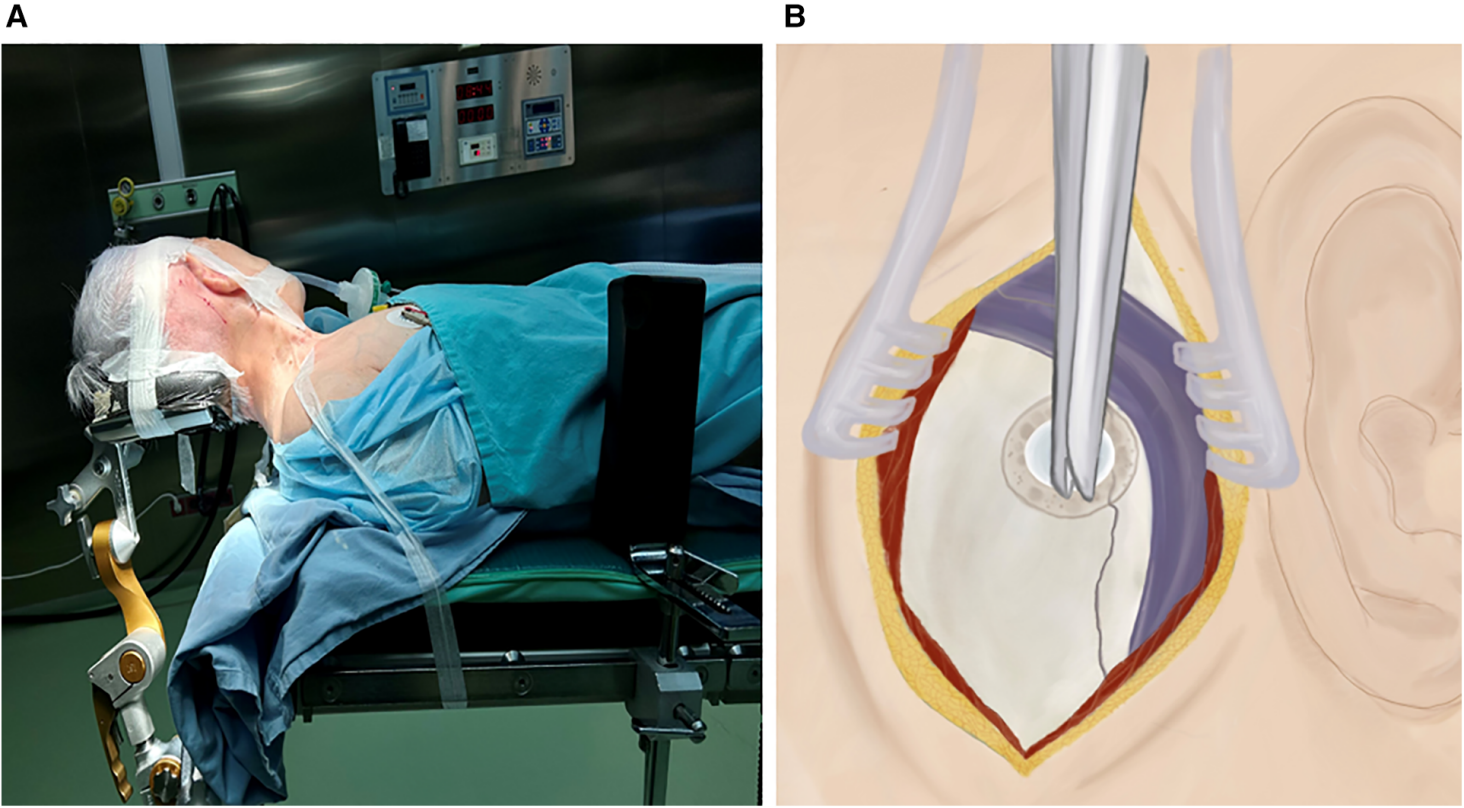
(**A**) Intraoperative view of patient supine positioning, (**B**) craniotomy.

Janetta's classic surgical technique involves positioning the patient laterally and fixating their head with 3-point Mayfield hardware at a slight 10-degree rotation away from the painful side (18, 19). Compared to the classic technique, our approach involves positioning the patient in a supine position with the head elevated above the body. We then flex and rotate the patient's neck to a contralateral angle of 45°–60° and support the head with a horseshoe-shaped headrest (Figure 2A). To ensure stability during the procedure, we use adhesive plaster to fix the patient's head onto the horseshoe-shaped headrest. Additionally, we tape down and secure the shoulder on the operative side, providing wider access to the surgical site. For further support and stability, the patient's body is secured to the operating table using a supporting frame.

### 3.2 Skin incision, craniotomy, and dural opening

A skin incision of approximately 5 cm in diameter is made 0.5 cm medially to the hairline. We also use a vertical linear incision behind the ear aligned with the hairline for our procedure (Figure 2B). Using monopolar cautery, we dissect the nuchal muscles and expose the wound using a Jansen retractor (18, 19).

**Figure 3:**
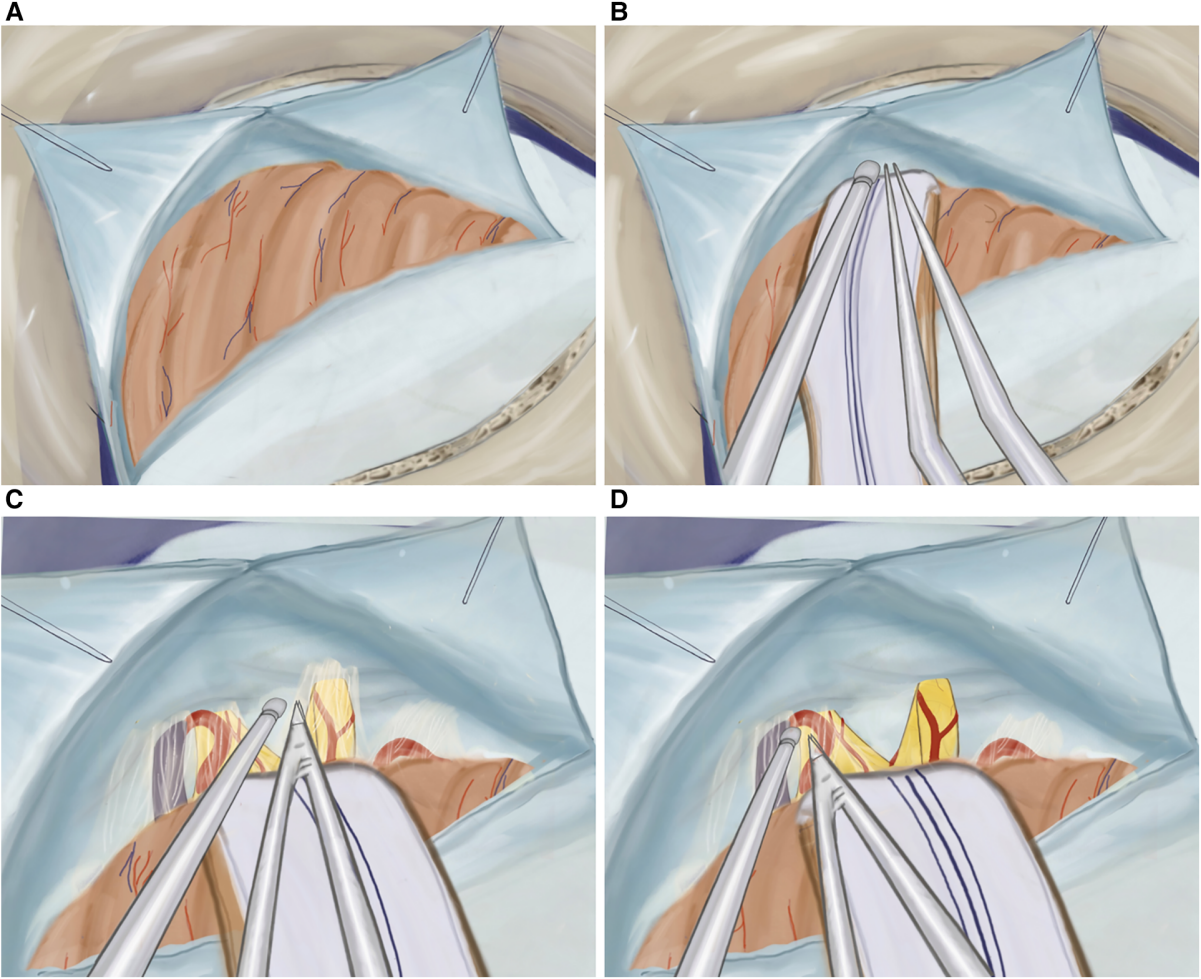
(**A**) Illustration of T-shaped dura opening, (**B**) retracted cerebellar hemisphere protected by cottonoids and rubber, (**C**) cisternal dissection of the facial and vestibulocochlear nerves, and (**D**) cisternal dissection of the trigeminal nerve.

At first, a single burr hole, measuring approximately 2.0 × 2.0 cm, is placed inferiorly to the connection point of the transverse and sigmoid sinuses (Figure 2B). This opening is then expanded either using Kerrison Rongeurs or by drilling through the mastoid bone while visualizing the sinuses. If the sinuses are injured during this process, hemostasis must be achieved using hemostatic agents such as Tachocomb, as coagulation should be prevented. In cases where the mastoid air cells are inadvertently opened during drilling, they must be sealed using wax to prevent postoperative nasal leakage. The dural opening into a “V” shape completely exposes the area between the angle of the transversal sigmoid sine 0.5 cm medially to the sinuses, which allows us to avoid significant cerebellar retraction (Figure 3A). Finally, we anchor the dural edge superiorly with Vicryl 5-0 suture (20).

### 3.3 Cisternal dissection and MVD plus technique

The cerebellar hemisphere is gently retracted using an aspirator and bipolar instrument while being protected by cottonoids and rubber (Figure 3B). A meticulous dissection of the arachnoid membrane is performed, and cerebrospinal fluid (CSF) is drained through aspiration creating more space in the cerebellopontine angle (Figure 3C).

The cranial nerves are identified, starting from the facial and vestibulocochlear nerves, emerging from the brainstem below, and laterally to the trigeminal nerve (Figure 3D). To prevent potential damage, such as facial paralysis or hearing impairment, a careful dissection of the arachnoid membrane of the cranial nerves is performed, avoiding excessive traction during the mobilization of the arachnoid. Additionally, before accessing the trigeminal nerve, the superior petrosal vein is identified and mobilized, which is normally located cranial and lateral to the trigeminal nerve.

**Figure 4:**
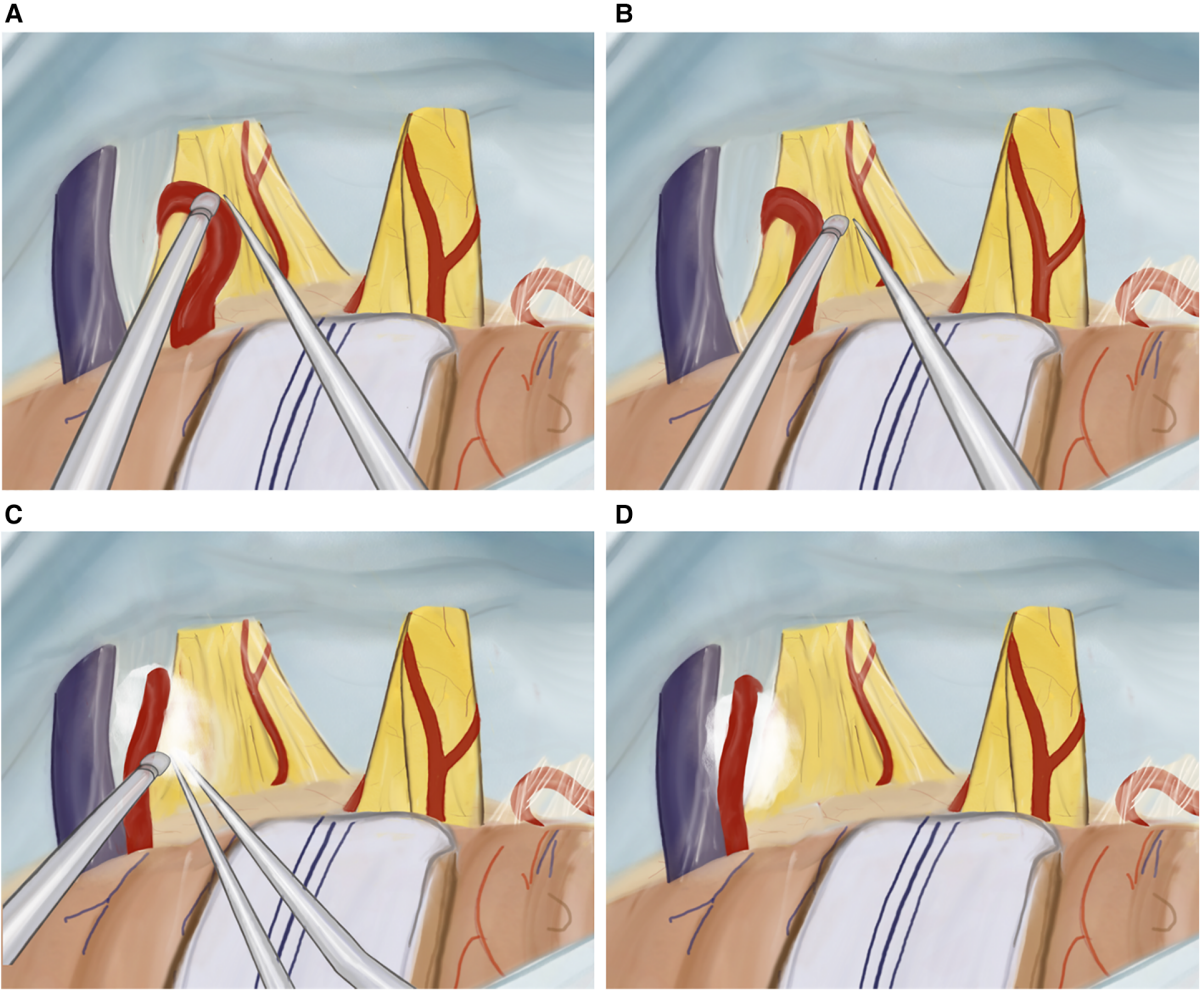
Illustration of MVD plus technique. (**A**) Mobilizing the artery from the trigeminal nerve, (**B**) internal neurolysis (combing) of the trigeminal nerve, (**C**) tephlon placement, (**D**) view after tephlon placement.

**Figure 5:**
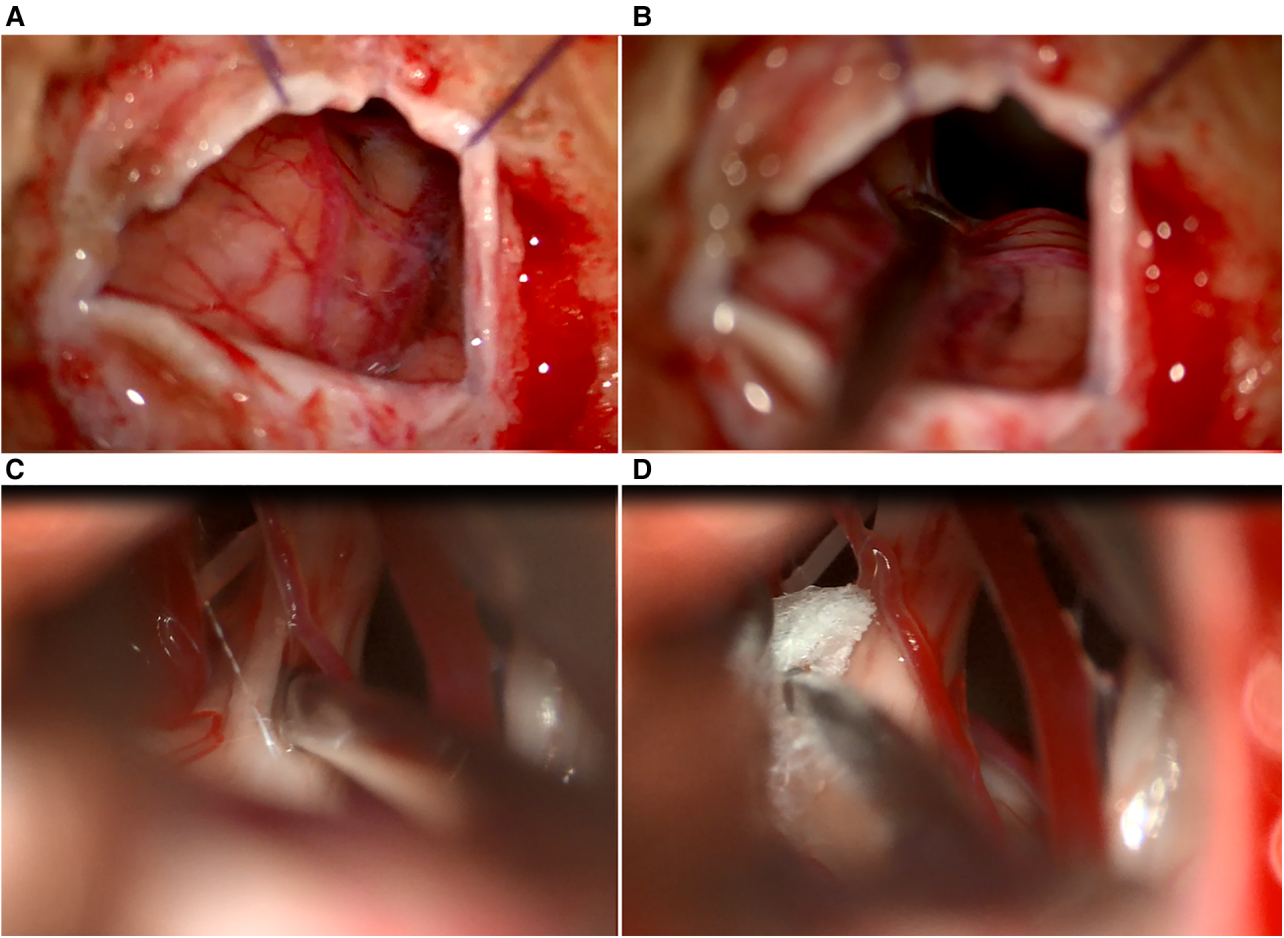
(**A**) Intraoperative view of dura opening, (**B**) retracted cerebellar hemisphere using an aspirator, (**C**) internal neurolysis (combing) of the trigeminal nerve, and (**D**) tephlon placement between the artery and trigeminal nerve.

Moving superiorly and anteriorly, the cisternal portion of the trigeminal nerve is identified. Any compression, whether arterial or venous, is relieved as neuro-vascular conflicts are dissected and mobilized away from the trigeminal nerve (Figures 4A,B). The surrounding arachnoid is carefully and precisely removed. Subsequently, a section of Teflon is prepared for use. We placed an appropriately sized Teflon cotton ball between the implicated blood vessel and the trigeminal nerve root, establishing a barrier that effectively prevents any further compression or contact between them (Figures 4C,D). Following MVD (Figures 5A,B), we perform the additional combing technique, as described by Ming-Xing Liu (21), which has demonstrated better results in comparison to MVD alone (Figures 5C,D).

Once the internal neurolysis (combing) of the trigeminal nerve is completed, the cisterns are thoroughly filled with a warm Ringer lactate solution. Before closing the dura mater, we confirm the proper placement of the prosthesis and ensure hemostasis is achieved. Even in the absence of vascular contact at the root entry zone, we still perform the arachnoid dissection and combing procedure as a preventive measure.

### 3.4 Wound closure and postoperative considerations

To seal the dura, fascia or muscle grafts are employed, and wax is applied to the bone to prevent any leakage of CSF. We then apply a layer of Gelfoam over the dura, followed by cranioplasty using methyl methacrylate. The muscle and subcutaneous layers are sutured separately, and the skin is closed using a continuous running technique with Vicryl 5-0 thread. This approach guarantees secure closure and promotes proper healing of the surgical site.

Following the surgery, patients were admitted to the intensive care unit for 3–4 h for close monitoring until they fully regained consciousness. They were then transferred to the standard ward, where they stayed for several days before being discharged home.

## 4 Results

### 4.1 Patient characteristics

This study involved 90 patients diagnosed with primary TN, with 56 females (62.22%) and 34 males (37.78%). The mean age of participants was 54.39 ± 12.23 years. The mean follow-up period was 32.78 ± 9.91 months, and all patients underwent MVD surgery. Among the patients, the majority presented with pain in both the maxillary and mandibular trigeminal nerve (40%), followed by pain in the maxillary trigeminal nerve alone (21.11%), and pain in both the ophthalmic and maxillary trigeminal nerve (20%). More than half of TN cases (65.56%) involved the right side. Overall, 83.33% of patients had neurovascular conflict and underwent Teflon placement in combination with nerve combing. The remaining 15 patients (16.67%) who did not have a neurovascular conflict underwent nerve combing alone.

Before the MVD, 90% of the patients were undergoing pharmacological treatments for TN. Specifically, 70.00% of them were being administered Carbamazepine, with dosages ranging from 200 to 2,000 mg. Additionally, 5.56% of the patients were prescribed polytherapy, involving the usage of multiple medications to manage the TN symptoms. Patient characteristics diagnosed with TN who underwent MVD are presented in Table 1.

**Table 1:** Characteristics of patients with trigeminal neuralgia.

Patients’ characteristic	Value, *N* (%)
Number of patients	90 (100)
Age (years, mean ± SD)	54.39 ± 12.23
25–49	28 (31.11)
50–59	34 (37.78)
60–69	20 (22.22)
70 and older	8 (8.89)
Sex	
Female	56 (62.22)
Male	34 (37.78)
Affected side	
Left	31 (34.44)
Right	59 (65.56)
Conflict	
Yes	75 (83.33)
No	15 (16.67)
Affected dermatomes	
V1	5 (5.56)
V2	19 (21.11)
V3	5 (5.56)
V1 + V2	18 (20.00)
V2 + V3	36 (40.00)
V1 + V2 + V3	7 (7.78)
Prior drug administration	
Carbamazepine	63 (70.00)
Gabapentin	2 (2.22)
Pregabalin	2 (2.22)
Ketoprofen	9 (10.00)
Polytherapy	5 (5.56)
No	9 (10.00)
Offending vessel	
Superior cerebellar artery	35 (38.89)
Anterior inferior cerebellar artery	8 (8.89)
Basilar artery	2 (2.22)
Transverse pontine vein	12 (13.33)
Vein of Dandy	10 (11.11)
Superior cerebellar artery + Transverse pontine vein	3 (3.33)
Superior cerebellar artery + Vein of Dandy	5 (5.56)
No	15 (16.67)
Surgical Intervention	
Teflon placement + Nerve combing	75 (83.33)
Nerve combing alone	15 (16.67)

### 4.2 Operative findings

During the MVD, neurovascular conflict was identified in 75 out of 90 patients (83.33%). Most of the neurovascular conflict cases (38.89%) were associated with the superior cerebellar artery. In 13.33% and 11.11% of cases, the offending vessels were the transverse pontine vein and superior petrosal vein, respectively (Table 1). The MVD procedures in this study were performed using a combination of Teflon placement and nerve combing in 83.33% of cases. The “MVD plus” method reduces the electrical sensitivity of irregular sensory trigeminal nerve fibers by disrupting their axonal connections. In cases where neurovascular conflict was not identified, trigeminal nerve combing alone was performed (16.67% of cases) (Table 1).

### 4.3 Complications

In this study population, no major complications were observed during the surgical procedure, with only one patient experiencing temporary leakage of CSF through the nasal passage. Three patients had pre-existing facial numbness, which did not show any significant change post-surgery, hence not considered operative complications. Transient facial numbness was observed in 18 patients, but it resolved within a duration of 1 week to 1 month. Two patients experienced hyperacusis, and two others reported hyperesthesia.

### 4.4 Follow-up

A follow-up with an average duration of 32.78 ± 9.91 months revealed that 80 patients (88.89%) achieved positive outcomes, as indicated by BNI scores I and II. The remaining 10 patients (11.11%) showed fair and poor scores of BNI III, IV, and V. Among these 10 patients, six reported effective pain management through medication, while the remaining four required radiofrequency thermocoagulation to address the symptoms. Out of the 15 patients who underwent nerve combing without Teflon placement, one patient reported no improvement after the surgery, while two others experienced a recurrence of pain. Patients with affected maxillary dermatomas and those with affected ophthalmic + maxillary dermatomas were more likely to experience fair + poor postsurgery BNI scores. Conversely, patients with neurovascular conflicts involving the maxillary + mandibular dermatomas demonstrated good BNI scores (*p* = 0.01). The follow-up data are given in Table 2.

**Table 2:** Outcomes of microvascular decompression for primary trigeminal neuralgia (mean follow-up period is 32.78 ± 9.91 months).

Variables	BNI score, *n* (%)		*P*-value
	Fair and poor (BNI III–V)	Good (BNI I and II)	
Total	10 (11.11)	80 (88.89)	
Age			0.39
25–49	3 (30.00)	25 (31.25)	
50–59	2 (20.00)	32 (40.00)	
60–69	4 (40.00)	16 (20.00)	
70 and older	1 (10.00)	7 (8.75)	
Sex			1.00
Female	6 (60.00)	50 (62.50)	
Male	4 (40.00)	30 (37.50)	
Affected side			0.48
Left	2 (20.00)	29 (36.25)	
Right	8 (80.00)	51 (63.75)	
Conflict			0.36
Yes	7 (70.00)	68 (85.00)	
No	3 (30.00)	12 (15.00)	
Affected dermatomes			0.01
V1	1 (10.00)	4 (5.00)	
V2	4 (40.00)	15 (18.75)	
V3	0	5 (6.25)	
V1 + V2	3 (30.00)	15 (18.75)	
V2 + V3	0	36 (45.00)	
V1 + V2 + V3	2 (20.00)	5 (6.25)	
Offending vessel			0.97
Superior cerebellar artery	4 (40.00)	31 (38.75)	
Anterior inferior cerebellar artery	1 (10.00)	7 (8.75)	
Basilar artery	0	2 (2.50)	
Transverse pontine vein	1 (10.00)	11 (13.75)	
Vein of Dandy	1 (10.00)	9 (11.25)	
Superior cerebellar artery + Transverse pontine vein	0	3 (3.75)	
Superior cerebellar artery + Vein of Dandy	0	5 (6.25)	
No conflict	3 (30.00)	12 (15.00)	
Surgical intervention			0.36
Teflon placement + Nerve combing	7 (70.00)	68 (85.00)	
Nerve combing alone	3 (30.00)	12 (15.00)	

## 5 Discussion

This study presents the outcomes of MVD intervention in patients with neurovascular compression syndromes, detailing the surgical technique employed. Overall, the study reveals good results in a study population with 80 out of 90 research subjects reporting BNI scores of I and II after the MVD.

Achieving good post-surgery outcomes depends on several factors, including ensuring the proper positioning of the patient. In this study, a supine position with appropriate head and neck support was employed. The position is considered for surgical procedures involving posterior fossa lesions (22) and ensures stability and facilitated surgical access. Furthermore, a supine position was reported to be effective for patients requiring close monitoring (23) as it is more physiologically natural for the patients. Furthermore, using a horseshoe-shaped headrest instead of the traditional three-point head fixation device allows for protection from potential neck or brachial plexus injuries resulting from over-rotation during surgical procedures (24).

Intraoperative management including small skin incisions, minimal craniotomy, and precise closure of the dura also influenced the results of MVD and subsequent post-surgery recovery. For skin incision and craniotomy with dural opening, Walter Dandy's use of a U-shaped incision is noteworthy. This technique helps prevent injury to the occipital artery and nerve, while also reducing the need for extensive muscle dissection below the posterior fossa (25). In line with the classic technique, our vertical linear retro sigmoid skin incision effectively maximizes exposure of the operative fields and minimizes the size of the craniectomy. This approach allows for smaller skin incisions with lengths of 5 cm, resulting in good post-surgery aesthetic results.

In this study, we perform a craniectomy averaging 4 cm square (20–20 mm) using bone rongeurs. This method is believed to help prevent CSF leakage by reducing the risk of opening the mastoid cells. In the rare event of mastoid cell opening, we follow the recommendation provided by Jannetta and colleagues, which involves closing them with bone wax (14, 15). Within our study population, only one patient experienced CSF leakage, accounting for 1.1%, which is relatively low in comparison with other study results that reported a CSF leakage occurrence from 2.3% to 2.8% (26, 27). CSF leakage is discouraged as it may lead to prolonged hospital stays with subsequent increased risk of infection and additional costs associated with treatment.

In our intradural and microsurgical steps, we do not commonly use lumbar catheters or Jannetta's cerebellar retractors for CSF drainage (18, 28, 29). Instead, we rely on microsurgical techniques to open the basolateral cisterns, which we believe sufficiently relaxes the cerebellum reduces the risk of complications associated with lumbar drainage, and allows CSF to naturally drain. This approach provides excellent visualization of neurovascular structures while minimizing the risk of harming the VII–VIII complex or cerebellum.

During the approach to the trigeminal nerve, preserving the superior petrosal vein (SPV) complex is crucial. While some suggest dissecting and coagulating the SPV (30), others advocate for preserving the SPV to maintain normal anatomy (31, 32). Our primary goal was to maintain natural anatomical structures. Consequently, we preserve the SPV complex in all cases.

The next key aspect of MVD involves the identification and mobilization of neurovascular conflicts between vessels and nerves. In cases of venous compression of the trigeminal nerve, the rate of recurrence is higher compared to arterial compression. According to Sumil K Nair's findings, patients experiencing TN due to venous compression generally present more severe pain outcomes following MVD, in contrast to patients solely affected by arterial compression (33). In our population study, cases with fair and poor outcomes could potentially be related to venous compression. In such cases, gamma-knife intervention is offered for patients as a treatment option. It should also be noted that identifying additional signs of compression, such as nerve thinning, arterial imprint, grooving, and distortion in the nerve course is important as some neurovascular conflicts may not present TN (34).

In terms of protective materials, the results of MVD show the same outcomes regardless of the material or placement technique (35–37), however, the position and amount of the protective material are important (38). Alternatively, another technique involves transposing the offending vessel from the overlying tentorium without interposing any foreign materials to achieve the same goal. These materials and techniques produce similar results (36, 37).

Following nerve decompression, we employ the “MVD Plus” Technique, which includes intraoperative neurolysis. Ming-Xing Liu's work demonstrates that combining sufficient microvascular decompression with nerve-combing for treating trigeminal neuralgia results in a high cure rate with fewer recurrences compared to microvascular decompression alone (21).

The use of meticulous methods, such as small skin incisions, minimal craniotomy, and the “MVD plus” technique has not only assisted prevent CSF leakage but has also improved post-surgery aesthetic outcomes and achieved good BNI scores, despite the absence of obvious conflicts.

## 6 Conclusions

In conclusion, the study found that patients with primary trigeminal neuralgia who underwent MVD had good BNI scores within this population. The outcome of the procedure depended on the affected dermatome of the trigeminal nerve with the vessel. Moreover, factors such as patient positioning, intraoperative management including small skin incisions, minimal craniotomy, precise closure of the dura, and intraoperative neurolysis may contribute to achieving positive clinical outcomes and satisfactory post-surgery aesthetics.

## Data Availability

The raw data supporting the conclusions of this article will be made available by the authors, without undue reservation.

## References

[1] Pearce JMS. Trigeminal neuralgia (Fothergill’s disease) in the 17th and 18th centuries. J Neurol Neurosurg Psychiatry. (2003) 74:1688. 10.1136/jnnp.74.12.1688 14638891 PMC1757428

[2] Cole CD Liu JK Apfelbaum RI. Historical perspectives on the diagnosis and treatment of trigeminal neuralgia. Neurosurg Focus. (2005) 18:E4. 10.3171/foc.2005.18.5.5 15913280

[3] Gerwin R. Chronic facial pain: trigeminal neuralgia, persistent idiopathic facial pain, and myofascial pain syndrome-an evidence-based narrative review and etiological hypothesis. Int J Environ Res Public Health. (2020) 17:7012. 10.3390/ijerph17197012 32992770 PMC7579138

[4] Headache Classification Committee of the International Headache Society (IHS). The international classification of headache disorders, 3rd edition. Cephalalgia. (2018) 38:1–211. 10.1177/0333102417738202 29368949

[5] Svedung Wettervik T Snel D Kristiansson P Ericson H Abu Hamdeh S. Incidence of trigeminal neuralgia: a population-based study in Central Sweden. Eur J Pain. (2023) 27:580–7. 10.1002/ejp.2081 36680398

[6] Zakrzewska JM Coakham HB. Microvascular decompression for trigeminal neuralgia: update. Curr Opin Neurol. (2012) 25:296–301. 10.1097/WCO.0b013e328352c465 22547101

[7] Bendtsen L Zakrzewska JM Heinskou TB Hodaie M Leal PRL Nurmikko T Advances in diagnosis, classification, pathophysiology, and management of trigeminal neuralgia. Lancet Neurol. (2020) 19:784–96. 10.1016/S1474-4422(20)30233-7 32822636

[8] Bendtsen L Zakrzewska JM Abbott J Braschinsky M Di Stefano G Donnet A European academy of neurology guideline on trigeminal neuralgia. Eur J Neurol. (2019) 26:831–49. 10.1111/ene.13950 30860637

[9] Araya EI Claudino RF Piovesan EJ Chichorro JG. Trigeminal neuralgia: basic and clinical aspects. Curr Neuropharmacol. (2020) 18:109–19. 10.2174/1570159X17666191010094350 31608834 PMC7324879

[10] Zakrzewska JM McMillan R. Trigeminal neuralgia: the diagnosis and management of this excruciating and poorly understood facial pain. Postgrad Med J. (2011) 87:410–6. 10.1136/pgmj.2009.080473 21493636

[11] Cruccu G Gronseth G Alksne J Argoff C Brainin M Burchiel K AAN-EFNS guidelines on trigeminal neuralgia management. Eur J Neurol. (2008) 15:1013–28. 10.1111/j.1468-1331.2008.02185.x 18721143

[12] Abdennebi B Guenane L. Technical considerations and outcome assessment in retrogasserian balloon compression for treatment of trigeminal neuralgia. Series of 901 patients—PubMed. Available online at: https://pubmed.ncbi.nlm.nih.gov/25101213/ (accessed April 10, 2024). 10.4103/2152-7806.137838 PMC4123256 25101213

[13] Régis J Tuleasca C Resseguier N Carron R Donnet A Yomo S The very long-term outcome of radiosurgery for classical trigeminal neuralgia. Stereotact Funct Neurosurg. (2016) 94:24–32. 10.1159/000443529 26882097

[14] Patel SK Markosian C Choudhry OJ Keller JT Liu JK. The historical evolution of microvascular decompression for trigeminal neuralgia: from Dandy’s discovery to Jannetta’s legacy. Acta Neurochir (Wien). (2020) 162:2773–82. 10.1007/s00701-020-04405-7 32519161

[15] Jannetta PJ. Arterial compression of the trigeminal nerve at the pons in patients with trigeminal neuralgia. J Neurosurg. (1967) 26(Suppl):159–62. 10.3171/jns.1967.26.1part2.0159 6018932

[16] Yang L Cheng H. Surgical technique management of microvascular decompression for trigeminal neuralgia. Ideggyogy Sz. (2022) 75:369–75. 10.18071/isz.75.0369 36541149

[17] Mizobuchi Y Nagahiro S Kondo A Arita K Date I Fujii Y Prospective, multicenter clinical study of microvascular decompression for hemifacial spasm. Neurosurgery. (2021) 88:846–54. 10.1093/neuros/nyaa549 33469667

[18] Jannetta PJ McLaughlin MR Casey KF. Technique of microvascular decompression. Technical note. Neurosurg Focus. (2005) 18:E5. 15913281

[19] Zhong J Li S-T Zhu J Guan H-X Zhou Q-M Jiao W A clinical analysis on microvascular decompression surgery in a series of 3,000 cases. Clin Neurol Neurosurg. (2012) 114:846–51. 10.1016/j.clineuro.2012.01.021 22310997

[20] da Silva OT de Almeida CC Iglesio RF de Navarro JM Teixeira MJ Duarte KP. Surgical variation of microvascular decompression for trigeminal neuralgia: a technical note and anatomical study. Surg Neurol Int. (2016) 7:S571–6. 10.4103/2152-7806.188916 27625893 PMC5009571

[21] Liu M-X Zhong J Xia L Dou N-N Shi J. Treatment of trigeminal neuralgia with “microvascular decompression plus” technique. J Neurol Surg B Skull Base. (2021) 82:e295–9. 10.1055/s-0040-1710520 34306952 PMC8289490

[22] Razak AA Youshani AS Krishnan R D’urso PI. Safety and feasibility of posterior fossa neurosurgery in the supine position—a UK series from a large centre. J Clin Neurosci. (2022) 101:150–3. 10.1016/j.jocn.2022.04.040 35597063

[23] Barrett WP Turner SE Leopold JP. Prospective randomized study of direct anterior vs postero-lateral approach for total hip arthroplasty. J Arthroplasty. (2013) 28:1634–8. 10.1016/j.arth.2013.01.034 23523485

[24] Shimizu K Matsumoto M Wada A Sugiyama T Tanioka D Okumura H Supine no-retractor method in microvascular decompression for hemifacial spasm: results of 100 consecutive operations. J Neurol Surg B Skull Base. (2015) 76:202–7. 10.1055/s-0034-1396660 26225302 PMC4433388

[25] Cohen-Gadol AA. Microvascular decompression surgery for trigeminal neuralgia and hemifacial spasm: naunces of the technique based on experiences with 100 patients and review of the literature. Clin Neurol Neurosurg. (2011) 113:844–53. 10.1016/j.clineuro.2011.06.003 21752534

[26] Herta J Schmied T Loidl TB Wang W Marik W Winter F Microvascular decompression in trigeminal neuralgia: predictors of pain relief, complication avoidance, and lessons learned. Acta Neurochir (Wien). (2021) 163:3321–36. 10.1007/s00701-021-05028-2 34674027 PMC8599248

[27] Lee H-S Cho K-R Park K Jeon C. Management of cerebrospinal fluid leakage after microvascular decompression surgery: clinical strategy. Life (Basel). (2023) 13:1771. 10.3390/life13081771 37629628 PMC10455648

[28] Masuoka J Matsushima T Kawashima M Nakahara Y Funaki T Mineta T. Stitched sling retraction technique for microvascular decompression: procedures and techniques based on an anatomical viewpoint. Neurosurg Rev. (2011) 34:373–9; discussion 379–380. 10.1007/s10143-011-0310-0 21347661

[29] Bond AE Zada G Gonzalez AA Hansen C Giannotta SL. Operative strategies for minimizing hearing loss and other major complications associated with microvascular decompression for trigeminal neuralgia. World Neurosurg. (2010) 74:172–7. 10.1016/j.wneu.2010.05.001 21300010

[30] Zhong J Li S-T Xu S-Q Wan L Wang X. Management of petrosal veins during microvascular decompression for trigeminal neuralgia. Neurol Res. (2008) 30:697–700. 10.1179/174313208X289624 18631430

[31] Tomasello F Esposito F Abbritti RV Angileri FF Conti A Cardali SM Microvascular decompression for trigeminal neuralgia: technical refinement for complication avoidance. World Neurosurg. (2016) 94:26–31. 10.1016/j.wneu.2016.06.097 27373414

[32] Narayan V Savardekar AR Patra DP Mohammed N Thakur JD Riaz M Safety profile of superior petrosal vein (the vein of dandy) sacrifice in neurosurgical procedures: a systematic review. Neurosurg Focus. (2018) 45:E3. 10.3171/2018.4.FOCUS18133 29961377

[33] Nair SK Xie ME Ran K Kalluri A Kilgore C Huang J Outcomes after microvascular decompression for sole arterial versus venous compression in trigeminal neuralgia. World Neurosurg. (2023) 173:e542–7. 10.1016/j.wneu.2023.02.090 36889635

[34] Maurya V Sreedhar CM Khera A Bhatia M Sharma V. Trigeminal neuralgia: when does neurovascular contact turn into a conflict? Med J Armed Forces India. (2019) 75:134–9. 10.1016/j.mjafi.2017.11.007 31065180 PMC6495106

[35] Pressman E Jha RT Zavadskiy G Kumar JI van Loveren H van Gompel JJ Teflon™ or ivalon®: a scoping review of implants used in microvascular decompression for trigeminal neuralgia. Neurosurg Rev. (2020) 43:79–86. 10.1007/s10143-019-01187-0 31786660

[36] Mitsos AP Georgakoulias N Lafazanos SA Konstantinou EA. The “hanging technique” of vascular transposition in microvascular decompression for trigeminal neuralgia: technical report of four cases. Neurosurg Rev. (2008) 31:327–30. 10.1007/s10143-008-0144-6 18470545

[37] Pines AR Butterfield RJ Turcotte EL Garcia JO De Lucia N Algier EJ Microvascular transposition without teflon: a single institution’s 17-year experience treating trigeminal neuralgia. Oper Neurosurg (Hagerstown). (2021) 20:397–405. 10.1093/ons/opaa413 33432975

[38] Ravina K Strickland BA Rennert RC Bakhsheshian J Russin JJ Giannotta SL. Revision microvascular decompression for trigeminal neuralgia and hemifacial spasm: factors associated with surgical failure. J Neurol Surg B Skull Base. (2019) 80:31–9. 10.1055/s-0038-1661348 30733898 PMC6365246

